# Treatment of Burns and Erysipelas by White Lead

**Published:** 1879-06

**Authors:** S. G. Riley

**Affiliations:** Shiloh, Ga.


### TREATMENT OF BURNS AND ERYSIPELAS BY WHITE LEAD.

By S. G. RILEY, M.D., Shiloh, Ga.

I was called to see Miss S—, æt. 30, who had been a subject of epilepsy from puberty. In one of her paroxysms she fell, face foremost, in the fire, and received an extensive burn of the face, neck, arms and breast. I arrived six hours after the accident and found the patient covered with poultices, and suffering extremely. A full dose of morphia and chloral was given at once, for the purpose of quieting the pain, and an examination made as to the extent of the injury. I found that the greater portion of the face, neck, upper part of back, breast and arms were severely and deeply burned, the skin in several places being baked brown, the right arm especially.

After the skin and adipose tissue came off, I could distinctly trace the course of the muscles of the arms and neck, and having seen white lead recommended for burns, by Dr. R. W. Westmoreland, in the Atlanta Medical Journal, and having met with great trouble in treating the accident, this being an extremely severe case, in which I saw but little hope of recovery, I concluded to give the lead recommendation a fair and impartial trial. I mixed the lead with linseed oil thoroughly, to about the consistency of thick cream, so that it could be spread with a knife-blade. On commencing the application, and after a portion of the burns had been covered, she urged me to proceed faster, as she was greatly relieved of the burning pain as fast as the lead was applied. I continued the application until a coating sufficient to exclude the air entirely was formed, which I think a very important point, and from this time she complained very little except with her right hand.

February 5.—Resting comfortably, with the exception of the right hand being greatly swollen, cold and painful.

February 7.—Has rested well. I removed the skin of right hand with the finger nails. She has lost all feeling in the hand, which looks very dark. I applied carbolic acid for disinfection purposes, the odor being very offensive. Gangrenous condition of the hand was marked, and the chances of saving it were hopeless.

February 9.—The burned surfaces suppurating freely; skin sloughing off; burns look healthy, except right hand, where a line of demarkation between dead and live tissue at wrist joint is marked.

February 11.—Resting well; bowels costive, for which I gave a simple laxative; I did not think it prudent to attempt amputation at present, as the action of the heart was very slow and feeble, the pulse ranging only from forty to fifty beats per minute. The soft parts of the nose having sloughed off the nostrils were completely plugged up, thereby rendering respiration in a manner impeded.

February 14.—Resting quietly; slowly but gradually improving up to 21st, at which time I asked for consultation, for the purpose of deciding upon the propriety of amputating the right hand. After a thorough examination it was thought extremely hazardous to attempt an amputation at the present time, as she would probably die from effects of an anæsthetic, or if she should survive this, she would die from shock. Then, too, the action of the heart still being feeble, and there being only the most distant shadow of a hope of her recovery, even after an operation, it was deferred. The patient continued to slowly improve up to the 15th of March, when in dresssing her, the hand dropped off at the wrist joint, and I removed several of the remaining small bones of the wrist. I would state, that she received but poor attention in nursing, and, being very poor, and mainly dependent on the cold charities of the neighborhood, could hardly obtain the actual necessaries of life, much less such a diet as a person in her condition actually required. I think to have treated this case by the old plan, she would have died in a very few days after the accident. The lead seemed to be very soothing, giving ease where large doses morphine and chloral failed. As the inflammation subsided, I discontinued the lead, and applied cloths thoroughly saturated with common axle grease, the burns healing kindly and rapidly.

May 20th.—Burns nearly all healed up; nature has given a very nice stump at the wrist joint, but the deformity about the face is very great, especially about the eyes. The under eyelids are entirely everted. The left corner of the under lip is contracted and misshapen, leaving the teeth exposed. The chin is drawn down towards the sternum, and the soft parts of the nose are destroyed. She is really a pitable looking object.

There is another disease in which I would be pleased to call attention to the benefit of the application of lead—namely erysipelas. This is a treatment that I have been using for the past fifteen years with entire success. I will give notes of two cases from my note book:

Case 1st, 1867.—Mrs. R. had an attack of erysipelas, resulting from a blister to the throat. It spread very rapidly over the face, arms and body, regardless of the application of tincture of iodine, nitrate silver, and all the usual and a great many of the unusual remedies. This lady had the advice of four physicians, who pronounced her case hopeless, and, as a dernier resort, therefore, I advised the application of the lead. Two hours after the first application was made, she was sleeping soundly. She awoke six hours afterward, being greatly refreshed, and, in two or three days, was convalescent without any other treatment.

In 1878, Mr. M. had erysipelas from a severe case of inflammatory sore eyes.

When I first saw the patient, in consultation, he had not slept any for thirty-six hours; he could not remain in one position two minutes at a time, the pain and burning rendering him so restless. His entire face and neck had been thoroughly painted with tincture iodine, and marked around with caustic, but the disease continued to spread. I advised the lead application, which after some hesitation on the part of patient, was agreed to under the promise that we would apply nothing to aggravate the burning. I assured him that it would not burn, but cure him, and that in one hour he would be sleeping soundly. Before the application was finished he was asleep and did not awake for eight hours. He had no more trouble with his erysipelas. I could give notes of several other cases but deem these sufficient. One fair and impartial trial will convince the most skeptical. I have never found it necessary to use any other treatment in erysipelas, and I have yet to meet with failure. A very important point is to apply the lead as thick as it can be spread with a knife-blade, so that the air may be entirely excluded. As a general rule, the patient will be relieved of suffering by the time the application is completed. The lead is physiologically extremely soothing to the inflamed parts, and is as sure a specific for this disease as morphine for a pain, or quinine for an intermittent, in my opinion.

Hoping these somewhat limited notes may be of interest to some, I will close with the promise of communicating to your journal whatever may be met with that I may consider interesting.

